
JOURNAL INFORMATION
==============================
NLM Title Abbreviation: Hist Cienc Saude Manguinhos
Journal ID: Hist Cienc Saude Manguinhos
NLM Journal ID: 9513999
Journal ID: hcsm

História, Ciências, Saúde - Manguinhos

ISSN: 0104-5970
EISSN: 1678-4758
Publisher: Casa de Oswaldo Cruz - Fiocruz

ARTICLE INFORMATION
==============================
PMCID: PMC10494970
DOI: 10.1590/S0104-59702023000100042
Article ID: 01501
Article version: 1
Subjects: Resenhas

Past and present at times of pandemic

Passado e presente em tempos de pandemia

http://orcid.org/0000-0003-0775-9928 Weber Beatriz Teixeira i
i Professora, Programa de Pós-graduação em História/Universidade Federal de Santa Maria. Santa Maria – RS – Brasil beatriztweber@gmail.com
Electronic publication date: 2023 Sep 4
Publication date: 2023
Volume: 30
Issue: Suppl 1
Electronic Location ID: e2023042
License: This is an Open Access article distributed under the terms of the Creative Commons Attribution License, which permits unrestricted use, distribution, and reproduction in any medium, provided the original work is properly cited.
License URL: https://creativecommons.org/licenses/by/4.0/

Figure count: 1
Table count: 0
Equation count: 0
Reference count: 3
Page count: NaN

==============================

Em 1695, o padre jesuíta Antônio Sepp ordenou uma sangria geral na redução de Nossa Senhora da Fé devido a uma peste de varíola hemorrágica. Dada a inexistência de instrumentos sangradores, músicos e ferreiros foram encarregados de produzir esses instrumentos. A sangria revelou-se pouco eficiente, levando o missionário a afirmar que os enfermos sentiam algum alívio mediante o uso de remédios caseiros e próprios da farmacopeia indígena, além de rigorosa dieta acompanhada de suco de limão e água fresca adoçada com mel. Esse episódio é um dos tratados pela experiente pesquisadora Eliane Cristina Deckmann Fleck sobre as epidemias registradas pela documentação jesuíta na América Platina. É uma das histórias apresentada no livro *Em tempos de pandemia*, publicação organizada a partir de dois ciclos de palestras em 2020, procurando refletir sobre o passado e o presente em tempos das incertezas da covid-19. As palestras foram organizadas pelo Grupo de Trabalho História e Saúde, da Associação Nacional de História, Seção Rio Grande do Sul (grupo dinâmico desde 2004), e o Programa de Pós-graduação em História da Unisinos, respondendo ao desafio que a pandemia criou. Os eventos contaram com diversos pesquisadores do Brasil e do exterior, vinculados a várias instituições. O livro apresenta as reflexões realizadas nos eventos e a partir deles.

A pandemia significou um crescimento nos estudos nessa área em todos os países, gerando muitas publicações, apesar de efetivamente poucas novidades terem surgido em termos da pesquisa (Hochman, Bird, 2021; Espindola et al., 2021). A iniciativa no Rio Grande do Sul, envolvendo vários outros lugares de produção de conhecimento, também atendeu a esse desafio de forma muito instigante. Os trabalhos de pesquisa apresentados foram desenvolvidos antes da pandemia, com reflexões bastante originais que tratam de múltiplos aspectos dessas histórias.

Os textos tratam de mostrar problemas que são muito recorrentes ao longo do tempo, como doenças epidêmicas, apresentando as situações vivenciadas por grupos sociais diversos e como essas situações foram enfrentadas em cada contexto. A demanda é por conhecimento do passado para ajudar a entender o evento pandêmico que enfrentamos. Os trabalhos tratam de enfermidades em outros momentos históricos, demonstrando que há relação com as percepções das doenças em outras épocas e as situações que cada período originava. As diferenças são ressaltadas porque cada contexto apresentou circunstâncias próprias, mas também indicam elementos comuns, como a estreita relação entre religiosidade e explicação das doenças.

Experiências de pesquisas diversas foram tratadas para dar visibilidade aos conhecimentos específicos sobre a enfermidade de cada grupo envolvido, à relevância das ideias religiosas, às incongruências entre uma modernização confusa e a insalubridade. Esses elementos procuram oferecer novas perspectivas para o contexto atual. Conforme o prefácio de Marcos Cueto, algo que as epidemias do passado têm em comum com a atual é magnificarem processos históricos de maior duração das desigualdades sociais. Os estudos do livro destacam a organização das ações de combate às doenças e as incongruências na organização dos serviços em vários momentos. Eles tornam claro como o Estado age com respostas emergenciais, ignorando as desigualdades sociais que amplificam os problemas. Eles tratam de como as ações vão se organizando ao longo do tempo, indo além da visão estrita do impacto ou de avaliações morais das doenças.

Todos os textos destacam como as enfermidades se propagavam facilmente devido à concentração de pessoas e à circulação existente entre as localidades, ora por navios, ora pelo contato entre grupos em terra. Todos evidenciam que as respostas às doenças foram viabilizadas por padrões de compreensão coletivos em cada época e lugar, com suas convenções e tradições compartilhadas, fosse pelos jesuítas que circulavam pela América, pelos moradores de diversas localidades, pelas autoridades que administravam medidas de contenção da doença. Todos os envolvidos com a experiência da doença compartilhavam conhecimentos com grupos locais, demonstrando uma interconexão de percepções.

Maior destaque foi dado a doenças como varíola, cólera e gripe espanhola, percebidas como momentos impactantes na organização da vida dos indivíduos, significativas para a compreensão de marcos na trajetória dos grupos sociais. Diversas fontes documentais foram utilizadas, como imprensa, legislação, relatórios, correspondências, demonstrando uma prática de pesquisa recorrente em todos os textos. Esse esforço de dimensionar trabalhos sobre doenças procurou oferecer aos leitores chaves de acesso à reflexão e à análise. Importante destacar que, apesar das proximidades, os textos demonstram como as situações são específicas, obedecendo a costumes advindos de tradições familiares ou religiosas, a partir dos quais os indivíduos construíram seus significados de acordo com as experiências que compartilharam.

De forma inovadora, os trabalhos destacam a importância das respostas populares em contextos extremos. Pensar como os envolvidos populares têm um papel de sujeitos na compreensão do adoecimento é fundamental para entendermos o desenvolvimento das doenças. A perspectiva dos indígenas, dos escravizados, dos moradores pobres das cidades mostra como as racionalidades populares têm um importante impacto no desenvolvimento de situações como a atual.

As abordagens tratam de aspectos que sempre tiveram pouca visibilidade na historiografia, como a morte para missionários jesuítas, as condições do cólera em 1855 e o papel na formulação de políticas de saúde pública, as manifestações da imprensa ilustrada sobre a gripe espanhola, e os bastidores de uma exposição sobre a gripe que precisou tornar-se virtual devido à pandemia.

Esse livro compartilha uma dinâmica de pesquisas com o público. São experiências que demonstram como esse grupo instiga-se a repensar seus temas de trabalho, especialmente numa situação de desafio. O livro estimula ainda mais o desafio que ele procurou enfrentar. Ainda há muito a ser pesquisado nessa área, sendo um fomento para novos olhares e para outros trabalhos. Frente às imensas incertezas da pandemia, o livro propõe-se a ajudar nessa nova busca.


REFERÊNCIAS

ESPINDOLA Mariola et al História, historiadores e a pandemia de covid-19 Topoi 22 48 588 621 2021
HOCHMAN Gilberto BIRN Anne-Emanuelle Pandemias e epidemias em perspectiva histórica: uma introdução Topoi 22 48 577 587 2021
KORNDORFER Ana Paula et al Em tempos de pandemia: reflexões necessárias sobre saúde e doenças no passado e no presente São Leopoldo Oikos 2021
